# Transformer-based convolutional forgetting knowledge tracking

**DOI:** 10.1038/s41598-023-45936-0

**Published:** 2023-11-04

**Authors:** Tieyuan Liu, Meng Zhang, Chuangying Zhu, Liang Chang

**Affiliations:** Guilin University of Electronic Technology, Guilin, 541004 China

**Keywords:** Computer science, Computational science

## Abstract

Knowledge tracking is to analyze the mastery of students' knowledge through the learning track. This is very important for online education, since it can determine a learner’s current knowledge level by analyzing the learning history and then make recommendations for future learning. In the past, the commonly used model for knowledge tracking is the convolutional neural network, but it has long-term sequence dependencies. With the invention of Transformer, it has excellent performance in long-sequence modeling by virtue of the attention mechanism, and is gradually introduced into the field of knowledge tracking. However, through our research, some knowledge tracking data sets have a large number of continuous and repetitive training, which will cause Transformer model to ignore the potential connections between some knowledge points. To overcome this problem, we introduce a convolutional attention mechanism to help the model perceive contextual information better. In addition, we simulate the forgetting phenomenon of students during the learning process by calculating the forgetting factor, and fuse it with the weight matrix generated by the model to improve the accuracy of the model. As a result, a Transformer-based Convolutional Forgetting Knowledge Tracking (TCFKT) model is presented in this paper. According to the experimental results conducted on the real world ASSITments2012, ASSISTments2017, KDD a, STATIC datasets, the TCFKT model outperforms other knowledge tracking models.

## Introduction

**Figure 1:**
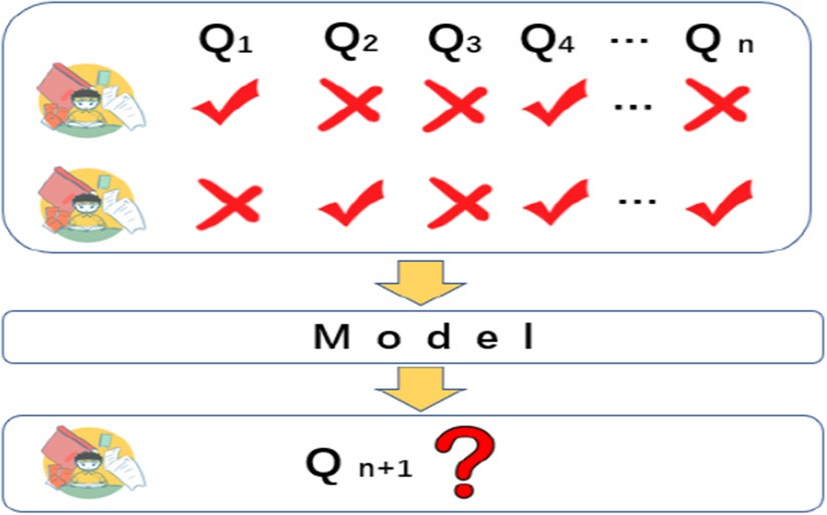
Knowledge tracking model.

With the continuous development of society, new challenges have been put forward for the cultivation of talents, which makes people pursue high-quality education while also continuously raising the high requirements for educational efficiency. It is hoped that within a limited time, students no longer limited by time and space, so as to reduce unnecessary time investment while learning high-quality knowledge, this has made online education develop rapidly in recent years.Compared with offline education in traditional education, online education has certain particularities. In offline education, teachers and students can communicate face to face and make accurate judgments on the degree of knowledge mastery. However, In the online education environment, students and teachers often communicate through the network environment, and the quality of education is often not as high as that of offline education. so in online education, as shown in Fig. 1, we can rely on the excellent performance of deep learning to establish knowledge tracking tasks for students' past learning trajectories, analyze the learning trajectories through models, and judge the degree of knowledge mastery of students.

Since the Google Translate team proposed the Transformer^1^ self-attention mechanism structure in 2017, it has received extensive attention in the fields of natural language processing and computer vision due to its excellent performance, and has gradually replaced the dominant position of the cyclic neural network model. Transformer plays an important role in time series prediction because it has no restrictions on the model's input and because the weight matrix generated by the attention mechanism can solve the problem of poor interpretability very well.However, through our analysis, it is found that due to the large number of continuous repeated training problems in some data sets, this will cause Transformer to assign too high weights to a small number of discontinuous learning records when calculating the weight matrix, which is to a certain extent It will affect the model to discover potential connections between different knowledge points. This paper^2^ serves as inspiration for us. The Transformer incorporates a convolutional neural network to improve the attention mechanism's perception of contextual information.

The issue of forgetting in the process of human learning has long been a focus of psychological inquiry. Ebbinghaus of the University of Berlin in Germany created a forgetting curve graph^3^ in 1885, indicating that human memory declines with time and that the rate of decline is not constant. In order to express the process of forgetting with specific numbers, After our analysis, we selected three features to calculate the forgetting probability. They are the interval time of the last training, the interval time of the same topic, and the interval time of the same knowledge point (there is a one-to-many relationship between topics and knowledge points, so the same knowledge points are often distributed in different topics).

Our contributions to this work can be summarized as follows:

In addition to the Recurrent Neural Network (RNN) model previously used by Deep Knowledge Tracing(DKT), we use the Transformer model as the foundation. This model can process sequences of any length, and the model is highly interpretable. The model's weight matrix can help us better understand the relationship between different knowledge points' associations.

To overcome the problem of the data set itself, we use a Convolutional Neural Network (CNN)^4^ to perform convolution processing on the input data, so that the attention mechanism can better perceive the potential connection between different knowledge points.

In addition to that, there is simulation of the forgetting behavior in the brain learning process, and use of the forgetting behavior as one of the reference standards for prediction, which is more in line with the human learning process.

## Related work

This section provides a brief overview of several knowledge tracking modeling methods that have been widely used in previous research.

The Bayesian Knowledge Tracing (BKT)^5^ model is one of the most widely used knowledge tracing models. BKT represents the learner's knowledge status as a set of binary variables, which represent whether students have mastered or not mastered a certain knowledge point. After each training session, BKT updates the probabilities of these binary variables using a Hidden Markov Model (HMM). BKT has been considered as the method of choice in the field of KT for the past 20 years, and improvements have been made on original models, such as variants of BKT^6^, variants of logistic regression^7^ and item response theory^8^, The performance difference between BKT and BKT variants is negligible^9^. Although BKT has had significant success in the field of KT, it also has significant problems. The state of students cannot be represented well by only binary variables^10^, and it can only model individual knowledge concepts, thus ignoring the relationship of different knowledge concepts.

Deep Learning Based Knowledge Tracing(DLKT), the DKT model proposed by Piech et al.^10^, is a pioneering work in the field of DLKT, as well as the basic model. RNN is the foundation of DKT. RNN is a memory-based sequence model, and the sequence structure allows it to conform to the recency effect in learning and preserve learning trajectory information^11^.RNNs including variants such as Long Short Term Memory^12^ (LSTM) and Gated Recurrent Unit^13^ (GRU) are the most widely used model in the field of DLKT. DKT takes students' learning interaction records as input and transforms them into a vector input model using one-hot encoding or compress sensing^14^. In DKT, the RNN's hidden state is interpreted as the student's knowledge state, and the hidden state is then passed through an activated linear layer to produce a series of prediction results. The length of the resulting sequence is equal to the number of questions, and each element represents the student's predicted probability of correctly answering the corresponding question.

Although DKT outperforms existing classical methods in terms of predictive performance, it has been criticized by a small number of other scholars due to its practicality in educational applications^15–19^. This is primarily due to the fact that the hidden state is inherently difficult to interpret as a knowledge state, and the DKT model does not conduct in-depth knowledge interaction analysis^20^, resulting in poor interpretability.

Context-Aware Attentive Knowledge Tracing(AKT)^21^, a model based on the self-attention mechanism, has achieved cutting-edge performance. This demonstrates the utility of self-attention mechanisms. Vaswani et al.^22^ later employed the self-attention mechanism instead. For RNN, the entire model framework is built, and the Transformer model is proposed. There is no long-term dependency issue because the Transformer model is independent of the RNN framework. Originally, the Transformer model was used for machine translation tasks, and it produced good results. Later, some researchers applied it to knowledge tracking and obtained results comparable to the DLKT model based on RNN, with no long sequence dependency problem.

Pandey et al.^23^ pioneered the use of the Transformer model in knowledge tracking, proposing the Self-attention Knowledge Tracing(SAKT) model. Choi et al.^24^ believed that the SAKT model's attention layer was too shallow and proposed the Deep Knowledge Tracing With Transformers(SAINT) model to address this issue. Pu et al.^25^ enhanced the Transformer's structure by including the structural information of the question and the time information of the answer.

## Model architecture

In this section, we will go over each component of the model in detail. Definitions for commonly used data for knowledge tracking are included. A positional encoding construct used to keep data position information safe. A demonstration of how and what convolutional layers do. How the attention mechanism works, as well as the weight matrix and forgetting factor.

### Problem definition

Generally speaking, the KT task can be defined in the following form: The student-learning interaction sequence is defined as ${X}_{t}=\left\{{x}_{1},{x}_{2},\cdots ,{x}_{t}\right\}$, where $t$ is the number of interactions, and each learning interaction is usually represented as a question–answer tuple $x_{t} = (q_{t} ,a_{t} )$, It means that the students answered the question $q_{t}$ at the moment, $a_{t}$ indicates the circumstances of the answer, ${a}_{t}\in \left\{\mathrm{0,1}\right\}$ indicates whether the question is answered correctly, 1 means correct answer, 0 means wrong answer, The model predicts that the probability of answering the next question correctly is $P(a_{t + 1} = correct|q_{t + 1} ,X_{t} )$.

### Position encoding

The RNN is a sequential structure, and the model already includes the positional relationship between the elements, but the Transformer is a completely different model than the RNN. It replaces the RNN with the Attention mechanism. As a result, the Transformer lacks position information, and the model is unable to determine the relative and absolute positions of each element in the sequence. Position information is critical in the knowledge tracking process, and the training position of knowledge points may be critical for prediction. Because the results are so important, we must include the positional relationship in the input vector so that the model can understand the positional information of the knowledge points in the input sequence.

**Figure 2:**
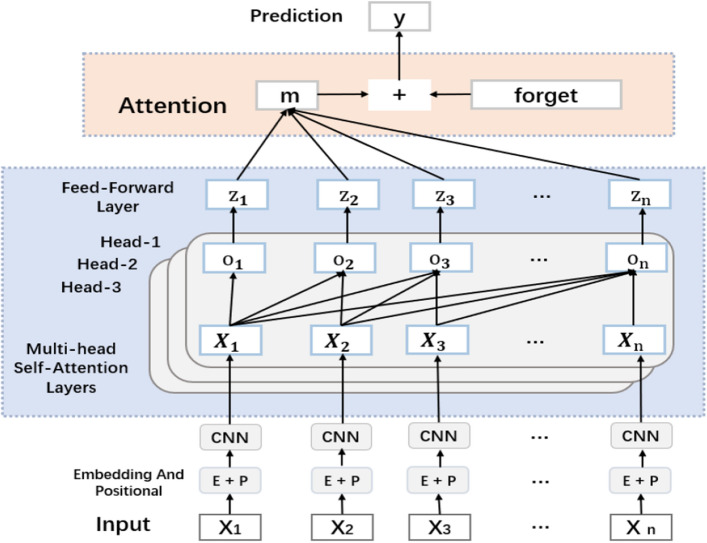
TCFKT model construction.

Figure 2 depicts the original method of the transformer we used for position encoding. In position encoding we give a history sequence of length n, where $\mathrm{i}$ represents for dimension, $\mathrm{k}$ represents a distance constant, $\mathrm{t}$ represents the position of each interaction in the history sequence, $\underset{{p}_{t}}{\to }\in {\mathbb{R}}^{d}$ represents the vector corresponding to the t position, and $\mathrm{d}$ is the dimension of the vector. $f:{\mathbb{N}}\to {\mathbb{R}}^{d}$ is a function of the production position vector $\underset{{p}_{t}}{\to }$, defined as follows:

1 $$\begin{array}{*{20}c} {\to _{{p_{t} }}^{\left( i \right)} = f\left( t \right)^{\left( i \right)} : = \left\{ {\begin{array}{*{20}c} {\sin \left( {\omega \cdot t} \right), if\; i = 2k} \\ {\cos \left( {\omega_{k} \cdot t} \right), if\; i = 2k + 1} \\ \end{array} } \right.} \\ \end{array}$$

where ${\omega }_{k}$ is defined as follows:

2 $$\begin{array}{*{20}c} {\omega_{k} = \frac{1}{{10000^{2k/1} }}} \\ \end{array}$$

The calculated position encoding can be combined with the original data encoding, so that the model can finally learn the dependencies between positions. The combination method is as follows:

3 $$\begin{array}{*{20}c} {X = Embedding\left( X \right) + Position} \\ \end{array}$$

### Convolutional layer

CNN has unique advantages in speech recognition and image processing with its special structure of local weight sharing. Its layout is closer to the actual biological neural network, and weight sharing reduces the complexity of the network.

**Figure 3:**
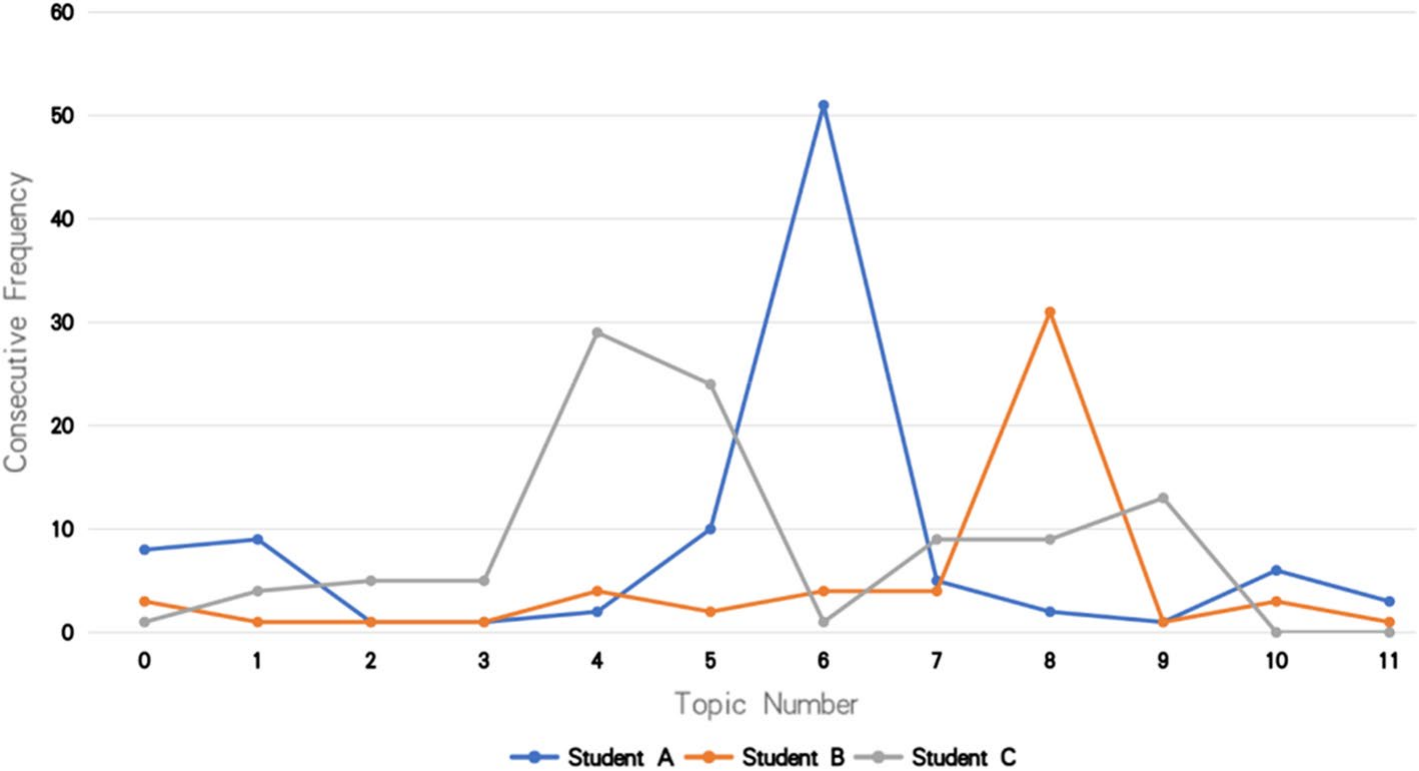
Student training records.

There are two main reasons for adding convolutional layers:

1. Through the analysis of the data set, we found that many students have trained a large number of the same knowledge points in a continuous period of time. We do not know whether this is caused by the fact that there are fewer knowledge points in the real data set, or the students' habit of doing questions. As shown in Fig. 3, Student A answered the 6th knowledge point 51 times in a row, Student B answered the 8th knowledge point 31 times in a row, and Student C answered the 4th knowledge point 29 times in a row.

2. The Transformer dot product operation has the problem of insensitive context awareness, it cannot pay attention to a small number of knowledge points that appear during the calculation process. For example, if a student learns 30 questions, the first 29 questions are the same addition operation, and the last question is multiplication operation. Transformer often ignores the relationship between the last question and the previous questions for this sequence, but this relationship should be noticed. Multiplication is a simplified addition, and they are related in the knowledge system.

In order to solve the above two problems, we first process the encoded data through the convolution layer, and then connect the processed data with the attention mechanism. The specific processing process is as follows.

4 $$\begin{array}{*{20}c} {X_{c} = \left( {X*W} \right)\left( {i,j} \right) = \mathop \sum \limits_{m} \mathop \sum \limits_{n} x\left( {i + m,j + n} \right)w\left( {m,n} \right)} \\ \end{array}$$

where $X$ is the input data and $W$ is the convolution kernel of the convolutional network.

### Attention mechanism

The Query, Key, and Value embedded vector concepts in Attention originate from the information retrieval system. Query (to match others): The input information has a guiding role, including what information we need, Key (to be matched): The content information represents other information to be matched, Value (information to be extracted): The information itself, V is just a simple expression information about the input features. Process the student’s input sequence ${X}_{t}$ to get ${X}_{c}$. ${X}_{c}$ is obtained by multiplying three different weight matrices $\left\{{W}^{Q},{W}^{K},{W}^{V}\right\}$ to obtain $\left\{Q,K,V\right\}$ as follows:

5 $$\begin{array}{*{20}c} {Q = X_{c} *W^{Q} } \\ \end{array}$$

6 $$\begin{array}{*{20}c} {K = X_{c} *W^{K} } \\ \end{array}$$

7 $$\begin{array}{*{20}c} {V = X_{c} *W^{V} } \\ \end{array}$$

After the input data is processed, the feature vector is obtained. First, the feature vector is convoluted, and the embedding vector Q, K, V is generated by the encoding process and then enters the Attention. First, the Attention score of each vector is calculated by the multiplication of the Q and K points. In order to ensure the stability of the gradient, it requires normalization of the Attention score. Next, the Attention score needs to be activated with the Softmax activation function, and the activation result is multiplied by V to obtain the weight matrix Z of each weighted input vector. Defined as follows:

8 $$\begin{array}{*{20}c} {Attention\left( {Q,K,V} \right) = softmax\left( {\frac{{QK^{T} }}{{\sqrt {d_{k} } }}} \right)V} \\ \end{array}$$

After the weight matrix is obtained, it will be sent to the feed-forward neural network layer. The first layer of the feed-forward neural network is the ReLU activation function, and the second layer is the Feed Forward Network(FFN). The specific definitions are as follows:

9 $$\begin{array}{*{20}c} {FFN\left( Z \right) = \max \left( {0,ZW_{1} + b_{1} } \right)W_{2} + b_{2} } \\ \end{array}$$

### Forgetting factor

One of the oldest problems in experimental psychology is forgetting. Ebbinghaus proposed the forgetting curve, which shows that people's forgetting changes with time, and that as time passes, the speed of forgetting decreases, eventually becoming a slow process with a relatively stable level. As a result, we examine students' forgetting behavior during the learning process using three variables: the interval time of the same knowledge point interaction, the interval time of the same topic interaction, and the interval time of the adjacent topic interaction (there is a one-to-many relationship between topics and knowledge points, so the same knowledge points are often distributed in different topics)..

Through the above three time factors, we will have forget behavior defined as:

$$\begin{array}{*{20}c} {f\left( {q,c,n} \right) = \mathop \sum \limits_{i = q,c,n} \theta_{q,q - 1} e^{{\Delta_{q,q - 1} }} + \theta_{c,c - 1} e^{{\Delta_{c,c - 1} }} + } \\ \end{array} \theta_{n,n - 1} e^{{\Delta_{n,n - 1} }}$$

**Figure 4:**
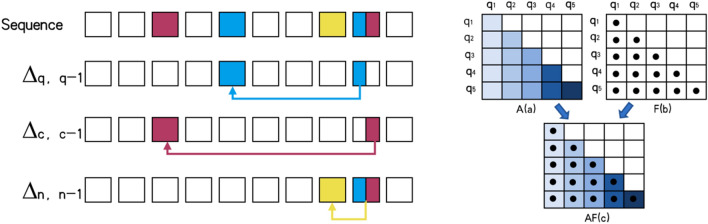
Forgetting factor principle.

$f\left(q,c,p\right)$ can be understood as the amount of forgetting of students at a certain moment, which consists of three parts of calculation, as shown in Fig. 4.

We define the interval between the current question and the previous same question as ${\Delta }_{n,n-1}$. The interval between the knowledge points contained in the current topic and the previous same knowledge point is ${\Delta }_{c,c-1}$, and the interval between the current topic and the previous topic is ${\Delta }_{q,q-1}$.

## Experiment

In this part, we selected 4 data sets in the real world to verify the effect of the model, and compared with three excellent models. The experimental results show that our model is better than other models in performance result.

### Dataset

As shown in Table 1, we conduct our experiments using four real-world public datasets: ASSISTments 2012, ASSISTments 2017^26^, KDD a^27^, and Statics.

**Table 1:** Experimental data.

	ASSISTments2012	ASSISTments2017	KDD a	Statics
Users	19,917	1709	569	4000
Skills	112	102	112	50
Questions	47,124	3162	574	618
Records	708,631	94,286	173,113	20,000

The ASSISTments dataset is the tutoring history gathered by online tutoring platforms, and it has long been one of the most commonly used datasets in the field of knowledge tracking. ASSISTments 2012 collected 708,631 learning records from 19,917 students. All records contained 112 knowledge points and 47,124 knowledge point-based questions. ASSISTments 2017 is the result of a competition between ASSISTment and 2017. A total of 942,816 interactions with 1,709 students have been recorded. Every record has 102 knowledge points and 3,162 questions made up of knowledge points. This is an interaction with the most informative dataset in terms of the number of interactions.

The KDD dataset contains rich skills and interaction information from interactions between students and the computer-aided tutoring system. KDD an is made up of 173,113 interaction records from 569 students, each of which contains 112 knowledge points and 574 questions.

The STATICS data set is a training record collected from university courses, which mainly includes the field of engineering mechanics. The data set records a total of 4,000 learners, 50 knowledge points, and 20,000 learning records of 618 questions.

### Result analysis

We also conducted a comparative study on the weight matrix generated by the Transformer to see if the CNN can improve the perception ability of the weight matrix to the knowledge points that appear in a small amount, as shown in Fig. 4.

**Figure 5:**
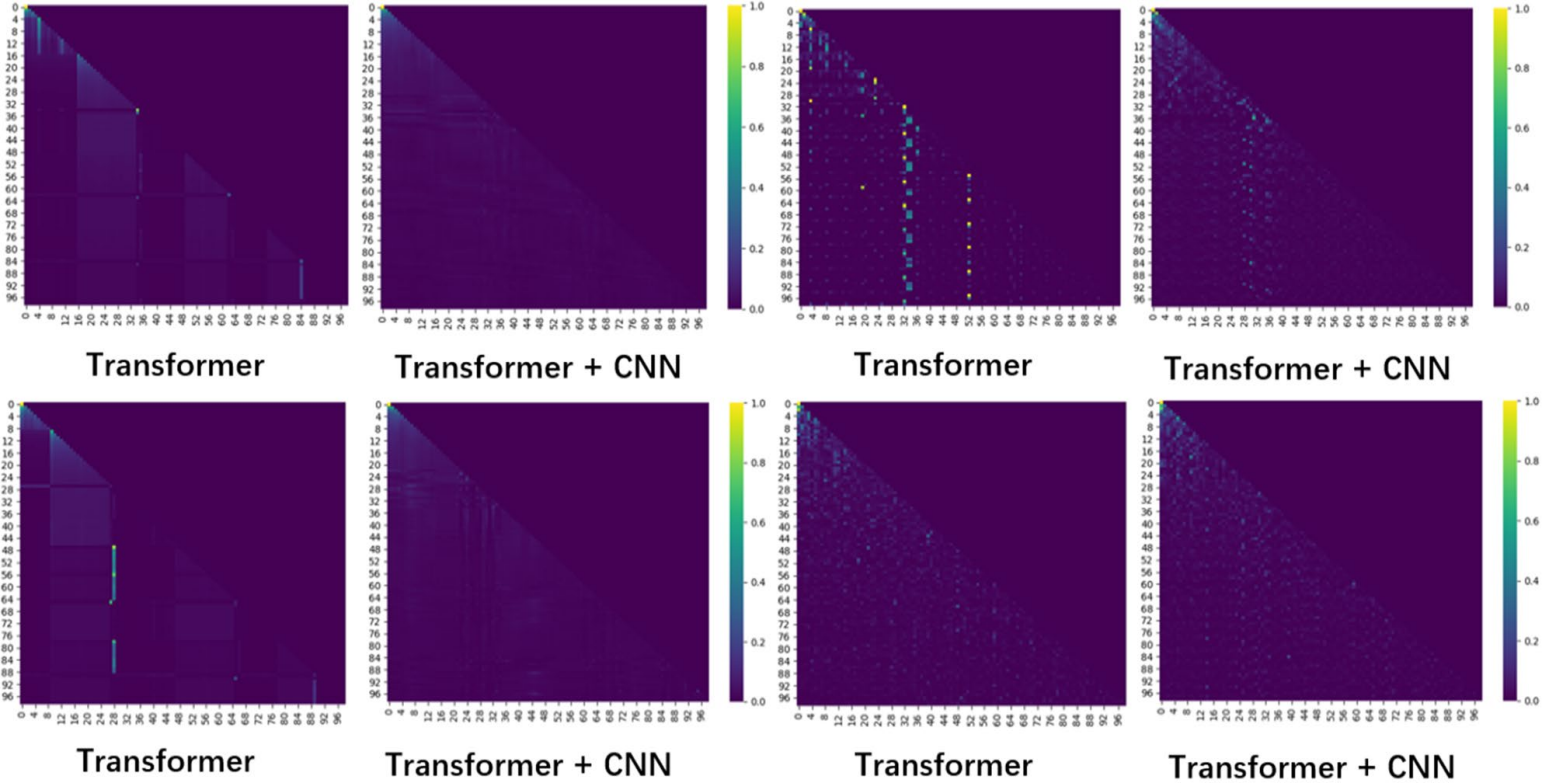
Comparison of weights before and after data convolution.

**Figure 6:**
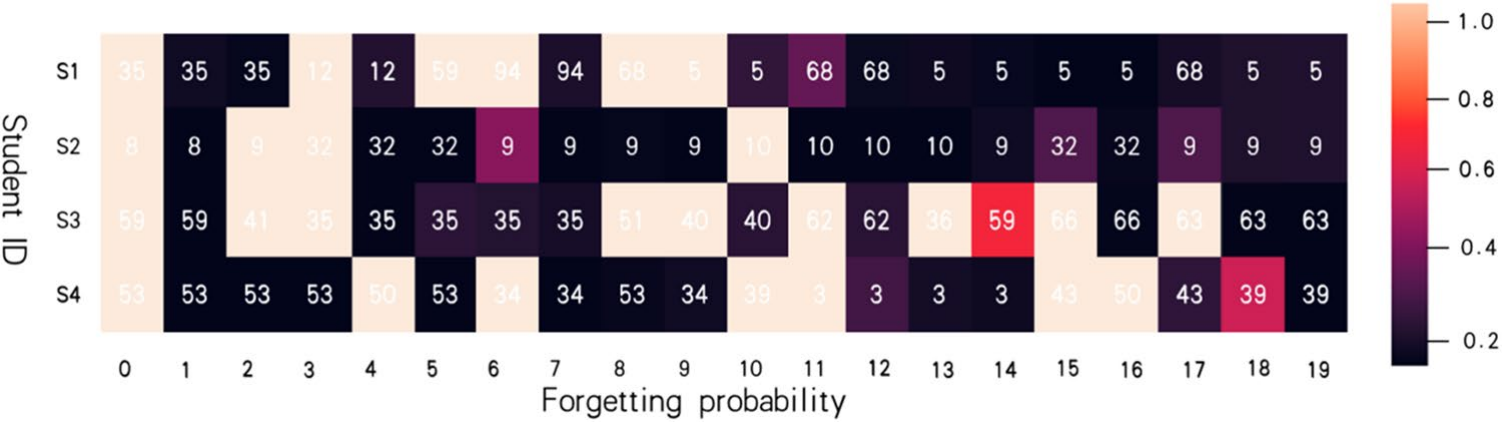
Forgetting factor calculation result.

We analyze the weight matrix in Fig. 5 by combining it with the original data. The knowledge point with very low frequency is the position in the visualization where the brightness is relatively unbalanced. The transformer's weight matrix cannot be well matched with other knowledge points. The weight distribution becomes more balanced after CNN fusion, indicating that after CNN processing, the correlation between knowledge points can be better discovered.To test whether our forgetting representation method can capture students' forgetting processes, we took 20 consecutive training records from the real data set and calculated the forgetting amount of each topic at each time point using our forgetting representation method. Figure 6 depicts the end result.Each row represents a student's learning record, and the forgetting probability is calculated by calculating the forgetting probability of the topics learned at each time point. Different colors represent different forgetting probabilities.

We can see from the analysis of the amount of forgetting that, using the students' record in the first row as an example, (1, 2) is the amount of forgetting calculated by the students for the first training of the ninth question. The color indicates that the amount of forgetting is relatively high. When the 9th question appears for the second time, the amount of forgetting decreases, and when the 9th question is retrained in a short period of time, the amount of forgetting decreases even more. This result is consistent with our acceptance of the law of forgetting. Furthermore, after calculating all of the forgetting amounts, we combine it with the weight matrix generated by the Transformer, as shown on the right in Fig. 6, to improve the model's prediction accuracy even further.

We run extensive experiments on real datasets to assess TCFKT's performance. In order to provide consistent experimental results, we evaluate the model's performance using the area under the curve (AUC) metric.

**Figure 7:**
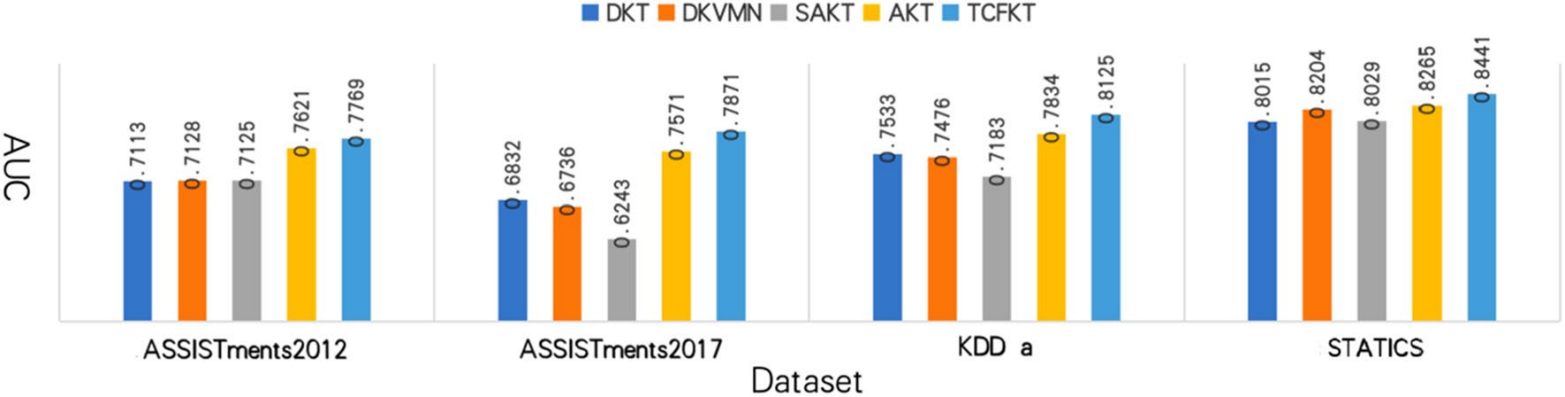
Experimental result.

In this experiment, we assess the reliability and progression of TFCKT by predicting whether students will answer correctly the next time based on previous learning sequences. Figure 7 depicts the experimental results. Several observations are as follows:

On all four datasets, our TCFKT model has the highest AUC. Our TCFKT model achieves a significant improvement of 3.96% on average to 0.7871 on the ASSIST17 dataset, compared to AKT's 0.7571^21^. In the KDD a dataset, the TCFKT model improves by 3.71% on average to 0.8125, compared to the 0.7834 achieved by AKT.On the Statics dataset, AKT and DKVMN^28^ have comparable performance, with 0.8265 and 0.8204, respectively, and TCFKT and AKT have a 2.12% improvement over DKVMN. The results of the ASSIST15 dataset show a significant improvement of 1.94% on average, with an AUC of 0.7621 for AKT and an AUC of 0.7701 for TCFKT, a significant improvement of 1.94% on average. On all four real datasets, TFCKT achieves the best prediction results. This result demonstrates that TFCKT can perform higher fusion of experimental data via CNN on the basis of Transformer, and further improve model accuracy via forgetting factor.

## Conclusion and future work

We propose a Transformer-based knowledge tracking model in this paper. We were initially inspired by the Transformer model. On this basis, we added a convolutional neural network to solve the problem of a large number of repetitions in students' problem records. The original model can overcome the context by using convolutional neural network data processing. It can also detect insensitive questions in order to better discover potential connections between various knowledge points. On this basis, the forgetting factor is added as a key factor. The forgetting factor simulates people's forgetting behavior during the learning process, and the output results of the remaining models are combined to better predict the results at the next time point. Finally, we run a large number of experiments on multiple real-world datasets, and the results show that our model is both interpretable and performant.

However, according to our findings, the learning order of students in knowledge tracking is a critical factor. The position encoding included with the Transformer model calculates the position of each interaction using a fixed sine and cosine, which may result in the position between the associated knowledge points being lost. As a result, we intend to use a complex position encoding method in the following step, so that the position encoding can contain more feature information.

## Data Availability

The datasets during the current study are available in the repository, [http://base.ustc.edu.cn/data/].

## References

[1] Vaswani, A., Shazeer, N., Parmar, N. *et al* Attention is all you need. *Adv. Neural Inform. Process. Syst.* 30, (2017).

[2] Li, S., Jin, X., Xuan, Y. *et al* Enhancing the locality and breaking the memory bottleneck of transformer on time series forecasting. *Adv. Neural Inform. Process. Syst.* 32, (2019).

[3] Murre JMJ, Dros J (2015). Replication and analysis of Ebbinghaus’ forgetting curve. PLoS One.

[4] Li, Z., Liu, F., Yang, W. *et al* A survey of convolutional neural networks: Analysis, applications, and prospects. *IEEE Trans. Neural Netw. Learn. Syst.* (2021). 10.1109/TNNLS.2021.3084827 34111009

[5] Corbett AT, Anderson JR (1994). Knowledge tracing: Modeling the acquisition of procedural knowledge. User Model. User-Adapt. Interact..

[6] Getseva, V., Kumar, A. N. Comparing Bayesian Knowledge Tracing Model Against NaĆÆve Mastery Model. *Intelligent Tutoring Systems*. (2021).

[7] Pavlik, P. I., Cen, H., Koedinger, K. R. Performance factors analysis: A new alternative to knowledge tracing. In *Proc of the 14th Int Conf on Artificial Intelligence in Education (AIED)*. 531-538 (Springer, 2009).

[8] Wauters K, Desmet P, Van Den Noortgate W (2010). Adaptive item-based learning environments based on the item response theory: Possibilities and challenges. J. Comput. Assist. Learn..

[9] Gong, Y., Beck. J. E., Heffernan, N. T. Comparing knowledge tracing and performance factor analysis by using multiple model fitting procedures. In *LNCS 6094: Proc of the 10th Int Conf on Intelligent Tutoring Systems (ITS)*. 35–44 (Springer, 2010).

[10] Piech, C., Bassen, J., Huang, J. *et al*. Deep knowledge tracing. In *Proc of the 28th Int Conf on Neural Information Processing System (NeurIPS)*. 505-513 (MIT, 2015).

[11] Khajah, M., Lindsey, R. V., Mozer, M. C. How deep is knowledge tracing. In *Proc of the 9th Int Conf on Educational Data Mining (EDM)*. 94–101 (IEDMS, 2016).

[12] Hochreiter S, Schmidhuber J (1997). Long short-term memory. Neural Comput..

[13] Weerakody PB, Wong KW, Wang G (2021). A review of irregular time series data handling with gated recurrent neural networks. Neurocomputing.

[14] Candès EJ, Wakin MB (2008). An introduction to compressive sampling. IEEE Signal Process. Mag..

[15] Wilson, K. H., Xiong, X., Khajah, M. *et al* Estimating student proficiency: Deep learning is not the panacea. In *Proc of the 27th Conf on Neural Information Processing Systems, Workshop on Machine Learning for Education*. [2020–10–22] (2016).

[16] Doleck T, Lemay DJ, Basnet RB (2020). Predictive analytics in education: A comparison of deep learning frameworks. Educt. Inf. Technol..

[17] Lalwani, A., Agrawal, S. Few hundred parameters outperform few hundred thousand. In *Proc of the 10th Int Conf on Educational Data Mining (EDM)*. 448–453 (IEDMS, 2017).

[18] Wilson, K. H., Karklin, Y., Han B. *et al* Back to the basics: Bayesian extensions of IRT outperform neural networks for proficiency estimation. In *Proc of the 9th Conf on Educational Data Mining (EDM)*. 539–544 (IDEMS, 2016).

[19] Ding, X., Larson, E. C. Why deep knowledge tracing has less depth than anticipated. In *Proc of the 12th Int Conf on Educational Data Mining (EDM)*. 282–287 (IDEMS, 2019).

[20] Lee, J., Yeung, D. Y. Knowledge query network for knowledge tracing: How knowledge interacts with skills. In *Proc of the 9th Int Conf on Learning Analytics & Knowledge (LAK)*. 491-500 (ACM, 2019).

[21] Ghosh, A., Heffernan, N., Lan, A. S. Context-aware attentive knowledge tracing. In *Proceedings of the 26th ACM SIGKDD International Conference on Knowledge Discovery & Data Mining*. 2330–2339[2020–10–29] (ACM, 2020).

[22] Vaswani, A., Shazeer, N., Parmar, N. *et al* Attention is all you need. In *Proc of the 31st Int Conf on Neural Information Processing Systems (NeurIPS)*. 6000-6010 (MIT Press, 2017).

[23] Pandey, S., Karypis, G. A Self-attentive model for knowledge tracing. In *Proc of the 12th Int Conf On Educational Data Mining (EDM)*. [2020–10–22] (IDEMS, 2019).

[24] Choi, Y., Lee, Y., Cho, J. *et al* Towards an appropriate query, key, and value computation for knowledge tracing. In *Proc of the 7th ACM Conf on Learning @ Scale (L@S)*. 341-344 (ACM, 2020).

[25] Pu, S., Yudelson, M., Ou, L. *et al* Deep Knowledge tracing with transformers. In *Proc of the 21st Int Conf on Artificial Intelligence in Education (AIED)*. 252–256 (Springer, 2020).

[26] Feng M, Heffernan N, Koedinger K (2009). Addressing the assessment challenge with an online system that tutors as it assesses. User Model. User-Adap. Inter..

[27] Stamper, J., Niculescu-mizil, A., Ritter, S. *et al* Challedge data sets from kdd cup (2010).

[28] Zhang, J., Shi, X., King, I. *et al* Dynamic key-value memory networks for knowledge tracing. In *Proceedings of the 26th international conference on World Wide Web*. 765-774 (2017).

